# Uterine Healing after Therapeutic Intrauterine Administration of TachoSil (Hemostatic Fleece) in Cesarean Section with Postpartum Hemorrhage Caused by Placenta Previa

**DOI:** 10.1155/2012/635683

**Published:** 2012-04-24

**Authors:** Katrine Fuglsang, Margit Dueholm, Estrid Stæhr-Hansen, Lone Kjeld Petersen

**Affiliations:** ^1^Department of Gynecology and Obstetrics, University Hospital of Aarhus, Skejby Sygehus, 8200 Aarhus N, Denmark; ^2^Institute of Pathology, University Hospital of Aarhus, Nørrebrogade, 8000 Aarhus C, Denmark

## Abstract

*Background*. Application of hemostatic fleece (TachoSil) directly onto the bleeding surfaces of the lower uterine segment has been used to obtain hemostasis during cesarean section caused by placenta previa. *Methods*. Eleven of 15 patients treated with TachoSil for excessive postpartum haemorrhage due to placenta previa were enrolled. An evaluation of the cesarean section scar by transvaginal ultrasound, the uterine cavity and endometrium by hysteroscopy, and the endometrium by biopsy were made. The main outcome measures were intrauterine adhesions, recovery of endometrium at the site of TachoSil application, visible remnants of TachoSil, and scar healing. *Results*. Eight patients had small remnants of TachoSil in the uterine cavity together with signs of resorption. All had a normal endometrial mucosa, and none had adhesions in the uterine cavity. All cesarean section scars were healed without defects. *Conclusion*. TachoSil did not seem to impair healing of the endometrium or scar formation in the uterus after intrauterine application. Resorption of TachoSil seems to progress individually. Intrauterine treatment with TachoSil is a valuable supplement to the traditional treatment of post partum haemorrhage and may help retain reproductive capability. This is a small study, and it will require more studies to confirm the reproducibility.

## 1. Introduction

The incidence of postpartum hysterectomy varies from 0.2 to 1.74/1000 deliveries. The most common indication for postpartum hysterectomy is abnormally adherent placenta (59%), 41% of which are due to placenta previa [1]. Delivery of patients with placenta previa is associated with the risk of excessive bleeding. Hysterectomy is a lifesaving procedure in the treatment of excessive bleeding due to placenta previa, but it deprives the woman of her ability for further reproduction [2]. In patients with placenta previa, traditionally methods, such as uterotonics, for reducing the blood loss [3] may be insufficient probably because of less muscular activity in the lower uterine segment [4]. Other methods are needed as alternatives to hysterectomy.

Application of TachoSil in the peritoneal cavity is a well-documented treatment, and studies show that the fleece is absorbed [5, 6]. Intrauterine treatment with TachoSil is a new technique with obvious short-term benefits to the patients, but its potential effects on uterine heeling are unknown, and insufficient healing and adhesions could impair the menstrual flow and decrease the ability to achieve future pregnancy. Moreover, the resorption of TachoSil in the uterine cavity is unknown.

The purpose of this study was to evaluate the potential side effects of the application of TachoSil in the uterine cavity. We studied the healing of cesarean section scars, resorption of TachoSil, recovery of the endometrium at the site of TachoSil and formation of adhesion in the uterine cavity. 

In this study, TachoSil was applied when the surgeon assessed the haemorrhage to be unacceptable despite uterotonics. We noted a trend toward faster use of TachoSil from the first patient to the last patient. Thus blood loss and the use of uterotonics and operative procedures to control haemorrhage were reduced [7].

## 2. Materials and Methods

Fifteen patients treated with TachoSil for excessive postpartum haemorrhage due to placenta previa in the period from January 1, 2007, to January 31, 2009, at the Department of Gynecology and Obstetrics, University Hospital of Aarhus, Skejby Sygehus, were invited for follow-up.

All had been diagnosed with partial or total placenta previa via ultrasound examination in gestational week 32. During their cesarean section, whether acute or elective, traditional uterotonics could not control the bleeding and the surgeon applied TachoSil, after which the hemorrhage was controlled.

The fifteen patients were offered an intrauterine examination and a check of their past gynecologic history. We were able to contact all 15: 11 agreed to participate and 3 declined because of ongoing pregnancy. The patients underwent a transvaginal ultrasound examination performed on a Voluson E8 Expert BT06 with a 12 MHz 3D/4D transvaginal transducer (GE Healthcare, USA). We evaluated the thickness of the endometrium, healing of the cesarean scar with presence of large uterine scars (<2.5 mm of remaining myometrium), and evidence of any remnants of the TachoSil. Hysteroscopy was performed with saline for distension and a 5 mm continuous flow office mini-hysteroscope (Bettocchi office hysteroscope, Karl Storz, Tuttlingen, Germany). Cervical canal, intrauterine cesarean section scar healing, and the endometrial mucus membrane were evaluated. Any remnants of the TachoSil and/or agglutination in the uterine cavity were also described. During the hysteroscopy, biopsies were obtained with a grasping forceps from the endometrium covering the lower uterine segment. One investigator with more than 10 years experience in ultrasound and hysteroscopy did all the examinations.

Two years after the clinical evaluation, a review of the 15 patients' hospital records was made to evaluate their number of pregnancies after treatment with TachoSil.

## 3. Results

Eleven patients participated in the study, and none experienced complications. Their mean age at delivery was 33.6 years (range 29–41). The mean time from the cesarean section to the examination was 11 months (range 5–25). 

There was an individual variation in the length of menostasia (1–8 months), but all had resumed menstruation (Table 1).

Six were primigravida and had not undergone any operative gynecological procedures. Five women had a history of previosly cesarean section. In this group the mean number of pregnancies was 3.6 (range 2–6) and the mean number of cesarean sections was 2.6 (range 2–4). One patient had a previous D&C, and one patient had been operated on twice for ectopic pregnancy. 

The ultrasound examinations visualized the incisional area after cesarean section in the anterior wall of the myometrium in all patients. The endometrium was described as thin at this location compared to the rest of the uterine cavity. In five of the patients, an echogenic density (punctate to 4 mm in diameter) gave the impression of a TachoSil remnant. In these patients, the mean time from cesarean section to the examination was 9.4 months (range 7–13) (Table 1).

**Figure 1:**
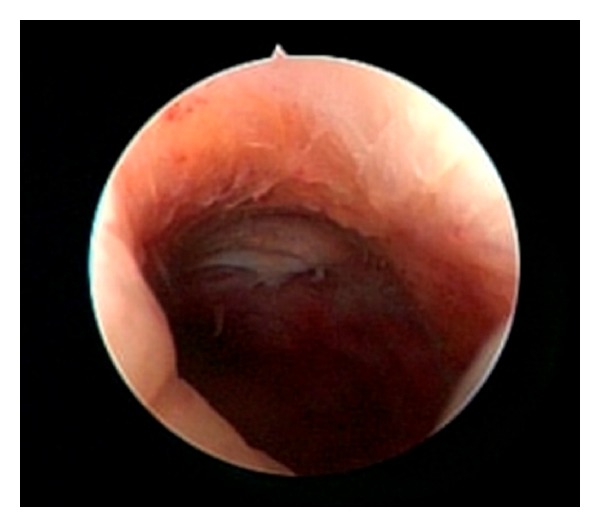
Intrauterine remnant of TachoSil (hemostatic fleece) visualized by hysteroscopy.

The hysteroscopy showed covering of the endometrium in the lower segment in all patients, and no adhesions were seen in the uterine cavity. In eight of the patients, TachoSil remnants were found (Figure 1). The size was described from punctate to 1.5 cm, and the size range was not consistent with the time from cesarean section to the examination (Table 1). 

If TachoSil remnants were found during the hysteroscopy, a biopsy was taken from this area, but otherwise random biopsies were taken. Unfortunately the biopsies were not useful for diagnostic purposes due to the size of the sample provided by the office hysteroscope. 

Six of the fifteen have spontaneously become pregnant (Table 1). Five have delivered at term and one is pregnant in gestational week 20 (Table 2). 

## 4. Discussion

Application of TachoSil at the lower intrauterine segment at cesarean section did not compromise the healing of the uterine scar or regeneration of the normal endometrium in the uterine cavity as evaluated by ultrasound or hysteroscopy.

Application of TachoSil is a simple, efficient, and rapid-onset treatment, giving control of bleeding. Consequently, none of the patients given TachoSil needed reoperation or developed post-partum endometritis [7]. Application of TachoSil to the lower segment does not give rise to formation of adhesions in the uterine cavity. Six patients became pregnant spontaneously, and all study patients examined had resumed menstruation and showed no signs of adhesion on hysteroscopy.

Delivery of patients with placenta previa may be difficult, and the associated risk of excessive bleeding can cause maternal death [8].

Using TachoSil to reduce the volume of haemorrhage during cesarean section due to placenta previa has demonstrated potential benefits. Thus this new method may control the heavy bleeding and save life without dramatic consequences for the woman giving birth. Therefore it seemed rational to introduce the procedure of applying TachoSil to the lower uterine segment in the presence of uncontrolled hemorrhage in spite of given uterotonics and before B-lynch suture or ligation of internal illiac artery was performed [7].

TachoSil consists of equine collagen, human thrombin, and fibrinogen. TachoComb, a predecessor to this product, is very similar, consisting of equine collagen, bovine thrombin, human fibrinogen, and bovine aprotinin. It is degraded intraperitoneally within 12 weeks in the majority of patients [5]. In a rabbit model, administration of TachoComb prevents adhesions compared to no treatment after operative procedures. Furthermore, histological investigations reveal that the outer surface of the applied TachoComb patch was completely covered by a new serosal layer 2 weeks after the operation [6]. These studies support the intrauterine appliance of TachoSil to avoid postpartum hysterectomy and preserve a woman's ability to become pregnant.

However, more studies are needed on the effect of TachoSil application in the uterine cavity; especially needed is an evaluation of the use of TachoSil in the corpus of the uterine cavity for placenta site bleeding after childbirth.

In four patients, there were no visible remnants of the TachoSil. In eight of the patients traces of TachoSil were found in the uterine cavity. Based on visual impression there were no endometrial reactions or signs of inflammation related to these remnants. The size of the TachoSil swab applied was 3.0 × 2.5 cm. Remnants varied from punctate to 1.5 × 1 cm, but our impression was that TachoSil was in the process of being resorbed in all patients. The largest remnant was found in patient 6, although TachoSil had been applied 13 months previously, confirming the individual variation in the time of resorption. The cesarean sectional scar healed without large scar defects. TachoSil has been used for the sealing of anastomoses [9] and in hernia repair [10], and, as evidenced by other studies, it does not seem to compromise scar formation.

## 5. Conclusion

Application of TachoSil to the lower uterine segment is an efficient haemostatic procedure to control excessive haemorrhage due to placenta previa. Technically the procedure is an easy and safe procedure and can be performed in all surgical wards. We did not find endothelial defects at the site of TachoSil application or the formation of adhesions in the uterine cavity. The fact that six of the patients became pregnant spontaneously supports this theory. Even though we found TachoSil remnants in eight of the patients, the size of these remnants supports the fact that the speed of resorption varies from patient to patient. Furthermore, this variation had no consequence for the women's reproductive ability, because all had resumed menstruation.

## Figures and Tables

**Table 1:** Results of clinical control and overview of pregnancies after treatment with TachoSil application in the uterine cavity.

Patient	Pregnancy	Cesarean section	Age	Menostasia	Time since delivery	Ultrasound		Hysteroscopy	Pathology	
				Month^a^	Month	Scar	TachoSil (mm)	TachoSil (mm)	Endometrium	TachoSil
No pregnancy after application of TachoSil in the uterine cavity										

2	6	2	41	Mens	25	Visible	no	no		
5	2	1	38	3	13	Visible	yes	yes
							2 × 1	punctual
6	4	3	32	Mens	13	Visible	no	yes 0	no	no
								15 × 1		
9	2	1	37	Mens	7	Visible	yes	yes
							3 × 3	3 × 3
10	1	0	34	6	7	Visible	yes	yes	yes	no
							4 × 2	4 × 2		
12	1	0	31	1	9	Visible	yes	yes	yes	yes
							punctuate	punctuate		
15	4	1	29	Mens	5	Visible	no	yes 0	yes	yes
								10 × 1		

Pregnancy after application of TachoSil in the uterine cavity										

3	1	0	31	^†^						
4	1	0	35	8	19	Visible	no	no	yes	yes
7	1	0	31	2	9	Visible	no	no		
8	1	0	30	1	11	Visible	yes	yes	yes	yes
							3 × 3	3 × 3		
11	1	0	32	3	6	Visible	no	yes punctuate	yes	no
13	1	0	31	^†^						

^a^Mens: menstruation was regained approximately two to three months postpartum, but they did not remember the exact time interval for menostasia.

^†^Did not attend the clinical control due to pregnancy.

**Table 2:** Outcome of pregnancies two years after clinical control.

Patient	Pregnancy	Outcome
3	1	Spontaneous vaginal delivery at term
4	1	Spontaneous vaginal delivery at term
7	1	Cesarean section at term due to previosly cesarean section with placenta accreta and previa
8	1	Cesarean section at gestational week 41+5
11	1	Spontaneous vaginal delivery at term
13	3	Abortus inhibitus
		Abortus provocatus
		Ongoing pregnancy in gestational week 20
